# Identification and characterization of a mirror-image oligonucleotide that binds and neutralizes sphingosine 1-phosphate, a central mediator of angiogenesis

**DOI:** 10.1042/BJ20131422

**Published:** 2014-07-24

**Authors:** Werner G. Purschke, Kai Hoehlig, Klaus Buchner, Dirk Zboralski, Frank Schwoebel, Axel Vater, Sven Klussmann

**Affiliations:** *NOXXON Pharma AG, Berlin, Germany

**Keywords:** aptamer, angiogenesis, sphingolipid, sphingosine 1-phosphate (S1P), Spiegelmer®, AEEAc, amino-ethyloxy-ethyloxy-acetyl, AMD, age-related macular degeneration, CHO, Chinese-hamster ovary, D-e, D-*erythro*, ERK, extracellular-signal-regulated kinase, FGF-2, fibroblast growth factor-2, GPCR, G-protein-coupled receptor, HUVEC, human umbilical vein endothelial cell, IGF-1, insulin-like growth factor-1, L-e, L-*erythro*, SK, sphingosine kinase, S1P, sphingosine 1-phosphate, S1PR, S1P receptor, VEGF-A, vascular endothelial growth factor-A

## Abstract

The sphingolipid S1P (sphingosine 1-phosphate) is known to be involved in a number of pathophysiological conditions such as cancer, autoimmune diseases and fibrosis. It acts extracellularly through a set of five G-protein-coupled receptors, but its intracellular actions are also well documented. Employing *in vitro* selection techniques, we identified an L-aptamer (Spiegelmer®) to S1P designated NOX-S93. The binding affinity of NOX-S93 to S1P had a *K*_d_ value of 4.3 nM. The Spiegelmer® shows equal binding to dihydro-S1P, but no cross-reactivity to the related lipids sphingosine, lysophosphatidic acid, ceramide, ceramide-1-phosphate or sphingosine phosphocholine. In stably transfected CHO (Chinese-hamster ovary) cell lines expressing the S1P receptors S1PR1 or S1PR3, NOX-S93 inhibits S1P-mediated β-arrestin recruitment and intracellular calcium release respectively, with IC_50_ values in the low nanomolar range. The pro-angiogenic activity of S1P, and of the growth factors VEGF-A (vascular endothelial growth factor-A), FGF-2 (fibroblast growth factor-2) and IGF-1 (insulin-like growth factor-1), was effectively blocked by NOX-S93 in a cellular angiogenesis assay employing primary human endothelial cells. These data provide further evidence for the relevance of extracellular S1P as a central mediator of angiogenesis, suggesting pharmacological S1P neutralization as a promising treatment alternative to current anti-angiogenesis approaches.

## Abstract 2

The mirror-image aptamer NOX-S93, identified by *in vitro* selection to bind specifically to S1P, inhibits signalling at S1P receptors. NOX-S93 inhibits the pro-angiogenic activity of S1P and other growth factors, revealing S1P neutralization as a promising anti-angiogenesis approach.

## INTRODUCTION

The sphingolipid D-e (D-*erythro*)-S1P (sphingosine 1-phosphate) is a signalling molecule with pleiotropic effects on proliferation, survival, migration and differentiation of diverse cell types [1]. It is generated by sphingosine kinases SK1 and SK2 from sphingosine, which is formed by ceramidase from ceramide and is degraded by several phosphatases and sphingosine lyase [2]. Whereas intracellular ceramide and sphingosine are pro-apoptotic, intracellular S1P acts in the opposite direction by stimulating cell growth and suppressing apoptosis. The balance of ceramide/sphingosine on one side and S1P on the other side determines whether a cell becomes apoptotic or survives [3]. The extracellular actions of S1P are mediated by binding to a set of S1P GPCRs (G-protein-coupled receptors) (S1PR1–S1PR5 or S1P_1_R–S1P_5_R) [4]. Accumulating evidence points to a role of S1P in angiogenesis of cancer and ophthalmic diseases such as wet AMD (age-related macular degeneration) [5]. Systemic S1P from the host has recently been found to promote cancer metastasis via S1PR2 signalling in a mouse xenograft model [6]. In fibrosis, a condition that is characterized by excessive production of extracellular matrix after injury, S1P seems to play a promoting role via ‘inside–out’ signalling at the S1PR2 [7]. The most prominent feature of S1P, the stimulation of immune cell egress from lymphoid organs into blood, mediated by signalling through S1PR1 [8], is a point of intervention for FTY720 (Fingolimod) a small molecule pro-drug that is approved for the treatment of multiple sclerosis. After phosphorylation *in vivo* by SK2 FTY720-phosphate (FTY720-P) eventually acts as a functional S1P antagonist by inducing down-regulation and degradation of S1PR1 [9]. Further promising strategies to antagonize S1P are related to the inhibition of the S1P-generating kinases SK1 and SK2. Safingol (L-*threo*-dihydrosphingosine), a substrate analogue of sphingosine that inhibits both kinases, is most advanced and has entered clinical studies for solid tumour treatment [10]. Another approach for intervention in S1P-related pathophysiology has been put into practice by a mAb (monoclonal antibody) that binds and neutralizes S1P and was shown to be effective in mouse xenograft and allograft tumour models [6,11] as well as in a mouse model of wet AMD, i.e. choroidal neovascularization [12]. The humanized version of this S1P-neutralizing antibody, sonepcizumab (aSONEP, iSONEP), has completed a Phase I study in patients with solid tumour (clinicaltrials.gov: NCT00661414) and is currently in Phase IIa trials in AMD patients (clinicaltrials.gov: NCT00767949, NCT01334255 and NCT01414153).

We were interested to explore the possibility whether an oligonucleotide-based structure, an aptamer, could also be able to bind the naturally occurring sphingolipid D-e-S1P with high affinity thereby inhibiting its biological activity. To achieve this, oligonucleotide libraries with up to 10^15^ different sequences are commonly screened directly against the given target molecule using the so-called SELEX process [13] to yield high-affinity aptamers [14]. For applications in a biological environment, however, aptamers have to be stabilized against nucleolytic degradation. Alternatively, the oligonucleotide libraries can be screened against the non-natural enantiomer of the given target [15], which is L-e (L-*erythro*)-S1P in this case. If the resulting aptamer is converted into its respective enantiomer (made from non-natural mirror-image L-nucleotides), this L-oligonucleotide (a so-called Spiegelmer®) binds to the natural target D-e-S1P. Owing to the mirror-image nature of the Spiegelmer® it is extremely stable in biological fluids and is also non-immunogenic. A number of Spiegelmer®-based compounds were identified in the past to bind and inhibit pharmacologically relevant peptides and proteins [16–19]. Currently, three Spiegelmers are in clinical development (Phase IIa) and have so far proved to be safe and well-tolerated [20–22].

In the present study, we report the identification of the high-affinity S1P-binding Spiegelmer® NOX-S93 and its *in vitro* characterization. The compound displays cross-reactivity to dihydro-S1P, but not to other related lipids. In cell-based systems NOX-S93 efficiently blocks S1P activity including its pro-angiogenic activities as well as those of other human growth factors such as VEGF-A (vascular endothelial growth factor-A), FGF-2 (fibroblast growth factor-2) and IGF-1 (insulin-like growth factor-1). Therefore NOX-S93 is a new modality to efficiently modulate S1P-related activities.

## EXPERIMENTAL

### Oligonucleotides and lipids

The non-natural enantiomer L-e-S1P(bio) [L-*e*-S1P, modified with biotin via an AEEAc (amino-ethyloxy-ethyloxy-acetyl)–AEEAc linker at C18 of the sphingolipid], was custom synthesized by Avanti Polar Lipids. As with the natural enantiomer D-e-S1P, directly biotinylated at C18, it was dissolved at 100 μM in 4 mg/ml essential fatty acid-free BSA (Sigma–Aldrich). However, the directly biotinylated D-e-S1P was first dissolved at 1 mM in hexafluoro-2-propanol (Merck Millipore) and dried down using air before it was redissolved in BSA. Other non-biotinylated natural enantiomeric S1P lipids (dihydro, C17, C18 and C20 Base), lysophosphatidic acid and sphingosylphosphorylcholine were first dissolved at 1 mM in methanol/H_2_O (95:5; v/v). After transfer into glass vials the solvent was removed using a stream of dry nitrogen. The residues were redissolved in PBS containing 4 mg/ml fatty acid-free BSA to 100 μM stocks. Sphingosine, ceramide and ceramide 1-phosphate were directly dissolved in ethanol to 10 mM, 10 mM and 1 mM stocks respectively. All lipids were from Avanti Polar Lipids with the exception of sphingosine which was purchased from Tocris.

The RNA library for *in vitro* selection with 34 internal random positions had the sequence 5′-GGAGCUUAGACA-ACAGCAGCGUGC-N_34_-GCACGCUCAGGUGAGUCGGUUC-CAC-3′. It was enzymatically generated using a single-stranded DNA library synthesized in-house as template for a one-cycle fill-in reaction with Vent exo^−^ DNA polymerase (New England Biolabs) and the T7 RNA polymerase promotor-containing forward primer 5′-TCTAATACGACT-CACTATAGGAGCTTAGACAACAGCAG-3′, followed by *in vitro* transcription with T7 RNA polymerase (Invitrogen). Reverse transcription was carried out with Superscript II (Invitrogen) and amplification with Vent exo^−^ DNA polymerase (reverse primer: 5′-GTGGAACCGACTCACCTGAG-3′). To follow the course of the selection, the RNA library was radioactively labelled with [γ^32^P]ATP (Hartmann Analytic) using T4 polynucleotide kinase (Invitrogen). Radioactivity was determined in a liquid scintillation counter (LS 6500; Beckman Coulter). D- and L-RNA (L-amidites were from Chem-Genes) were synthesized at NOXXON by standard phosphoramidite chemistry and the final Spiegelmer® candidate was conjugated to a 40 kDa polyethylene glycol moiety (JenKem) via an aminohexyl linker at the 5′-end [23] and named NOX-S93. Its sequence is 5′-(L)-GCGUGAAUAGCCGUUGAAACGCCUU UAGAGAAGCACUAGCACGC-3′. A Spiegelmer® with the reverse sequence (revNOX-S93), also PEGylated at the 5′-end, served as a control for the specificity of NOX-S93′s actions. Its sequence is 5′-(L)-CGCACGAUCACGAAGAG-AUUUCCGCAAAGUUGCCGAUAAGUGCG-3′. The under-lined nucleotides are deoxynucleotides.

### *In vitro* selection

The selection of S1P-binding aptamers was performed by incubating L-e-S1P(bio) with the RNA library in selection buffer [20 mM Tris/HCl (pH 7.4), 150 mM NaCl, 5 mM KCl, 1 mM MgCl_2_, 1 mM CaCl_2_, 0.1% Tween 20, 4 mg/ml BSA and 10 μg/ml yeast RNA]. Binding reactions were conducted in solution at 37°C for 14–16 h in the early and for 1–3 h in the later rounds. After the incubation time L-e-S1P(bio)–RNA complexes were immobilized on streptavidin- or neutravidin-coated beads (Thermo Scientific) and washed with selection buffer to get rid of non-binders and weak binders. Then the bound RNA was reverse transcribed and amplified. Beginning with round three, a counter-selection step with streptavidin- or neutravidin-coated beads was introduced before each selection reaction to avoid enrichment of the bead-binding aptamers. Also from round three onwards, in each round a control reaction without target was conducted in parallel to the binding reactions to ensure that binding signals were target-specific. The selection was started with a library complexity of ≈3.6×10^15^ molecules at concentrations of 10 μM RNA and 10 μM L-e-S1P(bio). During the selection the stringency was gradually increased by decreasing the concentration of the target and library and enhancing the washing intensity. After 16 rounds of selection, amplified DNA was cloned and sequenced (LGC Genomics).

### Pull-down assays for determination of affinity to S1P

The affinity of RNA to S1P was measured in a competition assay. Radioactively labelled RNA was incubated at 0.3–0.6 nM with a constant concentration of biotinylated S1P to accomplish 5–15% binding. After the addition of increasing amounts of non-labelled RNA and an incubation time of 2–3 h at 37°C in selection buffer the biotinylated S1P–RNA complexes were immobilized on streptavidin-coated beads, washed with selection buffer and the fraction of bound labelled RNA was determined in a scintillation counter. By plotting the fraction of bound labelled RNA against the concentration of non-labelled RNA, the dissociation equilibrium constant *K*_d(comp)_ was obtained using a four parameter fit (GRAFIT; Erithacus Software) assuming a 1:1 stoichiometry. To be able to include Spiegelmers into this assay, L-oligonucleotides were modified with two D–G nucleotides at the 5′-end and then labelled with [γ^32^P]ATP and T4 polynucleotide kinase.

### Post-selection modifications

Variants of the most affine Spiegelmer® (L-215-F9-002) were synthesized with single ribo- to deoxyribo-nucleotide exchanges and analysed in the pull-down assay using the radioactively labelled master Spiegelmer® L-215-F9-002 and an excess of the respective non-labelled Spiegelmer® variants. Positions with an increased affinity were combined.

### Inhibition of S1P signalling via S1PR1: blockade of β-arrestin recruitment

PathHunter™ eXpress growth arrested EDG-1 CHO-K1 (where CHO is Chinese-hamster ovary) β-arrestin GPCR cells (DiscoverX) were stimulated with S1P (10 nM). Upon activation, the interaction of S1PR1 (EDG-1) with β-arrestin leads to β-galactosidase enzyme fragment complementation, the activity of which was determined in the presence of different NOX-S93 concentrations. Approximately 10^4^ cells per well were seeded in a white 96-well plate with a clear bottom and cultivated for 48 h at 37°C and 5% CO_2_ in 100 μl of culture medium (DiscoverX). Stimulation solutions (10 nM S1P plus various concentrations of Spiegelmer®) were made up as 11-fold concentrated solutions in HBSS (Hanks balanced salt solution; Gibco) supplemented with 1 mg/ml BSA and 20 mM Hepes, mixed thoroughly and incubated at 37°C for 30 min. Stimulation solution (10 μl) was added per well (triplicates) and cells were incubated for 90 min at 37°C and 5% CO_2_. For quantification of β-galactosidase activity, 55 μl of Working Detection Reagent Solution (DiscoverX) was added and incubated for 90 min at room temperature. Luminescence was subsequently measured in a Fluostar Optima multidetection plate reader (BMG), corrected for background and the maximum luminescence signal was set to 100%. The IC_50_, i.e. Spiegelmer® concentration at half-maximal luminescence, was calculated using non-linear regression (four parameter fit) with Prism 5.04 (GraphPad) software.

### Inhibition of S1P signalling via S1PR3: blockade of intracellular calcium release

A stably transfected cell line expressing human S1PR3 was generated by cloning the sequence coding for human S1PR3 (NCBI accession number NM_003226) together with the sequence coding for the human G-protein G_α15_ (NCBI accession number M63904) into a plasmid vector suitable for eukaryotic expression (pVITRO2; Invivogen). CHO-K1 cells (DSMZ), adapted to grow in serum-free UltraCHO medium (Lonza), were transfected with the S1PR3-G_α15_ plasmid and stably transfected cells were selected by treatment with blasticidin.

Approximately 5×10^4^ S1PR3- and G_α15_-expressing cells per well were seeded in a black 96 well-plate with a clear bottom and cultivated overnight at 37°C and 5% CO_2_ in UltraCHO medium which contained in addition 100 units/ml penicillin, 100 μg/ml streptomycin and 10 μg/ml blasticidin. The stimulation solutions (10 nM S1P plus various concentrations of Spiegelmer®) were made up as 10-fold concentrated solutions in UltraCHO medium containing 20 mM Hepes and 5 mM probenecid (CHO-U+) in a 96-well ‘low profile’ PCR plate. Before loading with the calcium indicator dye FluoForte (Enzo Life Sciences), cells were washed once with 200 μl of CHO-U+. Then 90 μl of the indicator dye solution [5.56 μg/ml FluoForte and 0.044% pluronic 127 (Invitrogen) in CHO-U+] was added and the cells were incubated for 60 min at 37°C. After background fluorescence measurement at an excitation wavelength of 485 nm and an emission wavelength of 520 nm in a Synergy2 (BioTek) multidetection plate reader 10 μl of the stimulation solutions were added. The measured fluorescence signal was corrected for background fluorescence and the maximum fluorescence signal was set to 100%. The Spiegelmer® concentration at which the rise of intracellular calcium was blocked by 50% (IC_50_) was calculated using non-linear regression (four parameter fit) with Prism 5.04 software.

### Test of cross-reactivity to S1P-related lipids

Cross-reactivity of NOX-S93 to S1P-related lipids was tested in the calcium-release assay in direct or competitive mode depending on each lipid's activity in the S1PR3-expressing cell line. C17-S1P stimulated as effective as S1P (10 nM), whereas C20-S1P, dihydro-S1P and lysophosphatidic acid were used at 80 nM, 30 nM and 50 nM respectively, to stimulate the rise of intracellular calcium. Therefore cross-reactivity of the Spiegelmer® could be tested in a direct mode such as the measurement of IC_50_ to S1P. Cross-reactivity to sphingosine, ceramide, ceramide 1-phosphate and sphingosylphosphorylcholine, however, was tested in a competitive mode. In the competitive mode, cellular activation with 10 nM S1P was blocked by 100 nM NOX-S93. Test lipids were added in excess to compete for the inhibitory action of NOX-S93 on S1P-induced S1PR3 signalling.

### Inhibition of HUVEC spheroid sprouting in the cellular angiogenesis assay

The ability of NOX-S93 to inhibit angiogenesis was tested in a spheroid-based cellular 3D angiogenesis assay (ProQinase). Primary HUVECs (human umbilical vein endothelial cells; Promocell) were grown in endothelial cell growth and basal medium (ECGM/ECBM; Promocell) at 37°C and 5% CO_2_. After 3–4 passages spheroids were prepared by pipetting 500 HUVECs in a hanging drop on plastic dishes to allow overnight spheroid aggregation [24]. A total of 50 HUVEC spheroids were then seeded in 0.9 ml of a collagen gel and pipetted into individual wells of a 24-well plate to allow polymerization. Sprouting was induced after 30 min with either 100 nM S1P (Otto Nordwald), 0.65 nM human VEGF-A_165_ (ProQinase), 1.5 nM human FGF-2 (R&D Systems) or 65 nM human IGF-1 (Sigma–Aldrich) plus various concentrations of NOX-S93 (0.01–10 μM) by pipetting 100 μl of a 10-fold-concentrated working solution on top of the polymerized collagen gel. Plates were incubated at 37°C for 24 h and fixed by the addition of 4% Roti-Histofix (Roth).

Sprouting intensity of HUVEC spheroids was quantified using an image analysis system determining the cumulative sprout length per spheroid using an inverted microscope and the digital imaging software Analysis 3.2 (Soft imaging system). The mean of the cumulative sprout length of ten randomly selected spheroids was analysed as an individual data point.

IC_50_ determination was done using the Prism 5.02 software with constrain of the bottom (median of basal sprouting) to 0 and top (median sprouting induced by the respective growth factor) to 100% using non-linear regression (four parameter fit).

### Inhibition of ERK1/2 phosphorylation

HUVECs were seeded in six-well plates with a density of 1.8×10^6^ cells per well in EBM-2 medium with supplements (Lonza). The next day medium was changed (EBM-2 with 0.2% FBS) and the following day cells were serum starved for 3 h before stimulation. NOX-S93 or revNOX-S93 were pre-incubated for 20 min with 100 nM S1P. HUVECs were stimulated for 5 min and subsequently lysed in lysis buffer [20 mM Tris/HCl (pH 7.5), 150 mM NaCl, 1 mM EDTA and 1% Triton X-100] including PhosStop phosphatase inhibitor (Roche Diagnostics). Protein concentration was determined using the bicinchoninic acid method (Thermo Scientific). Protein lysates (5 μg) from each treatment were separated on 10% Novex® Tris Glycin gels (Invitrogen), transferred on to Amersham Hybond™ PVDF membranes (GE Healthcare) by blotting and probed with a phosphospecific anti-ERK1/2 (extracellular-signal-regulated kinase 1/2) antibody (Cell Signaling Technology) using the Snap i.d. detection system (Merck Millipore) in accordance with the manufacturer's recommendations.

## RESULTS

### Identification of a high affinity D-e-S1P-binding Spiegelmer®

**Figure 1:**
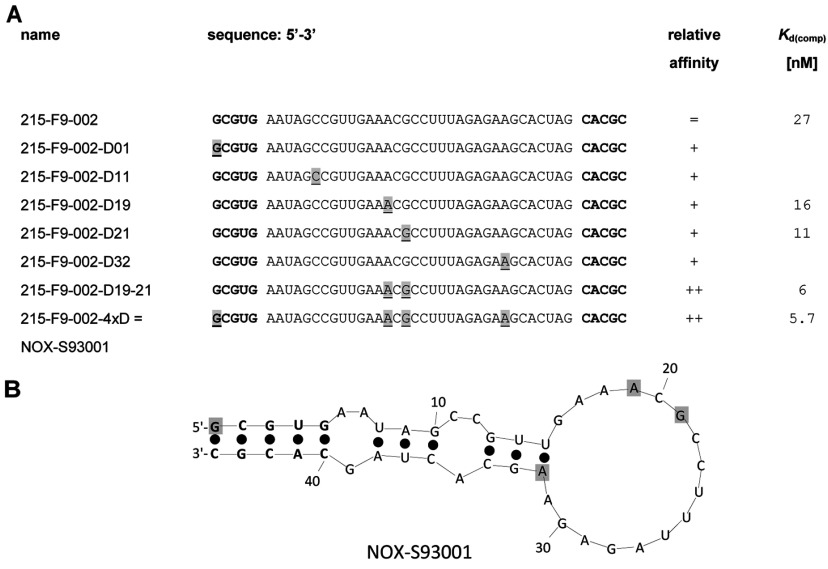
S1P-binding sequence variants and lead Spiegelmer® NOX-S93 (**A**) The master sequence 215-F9–002 and its variants as well as their relative affinities and *K*_d_ values. (**B**) Secondary structure prediction of NOX-S93001. Deoxyribonucleotides are shaded in grey.

**Figure 2:**
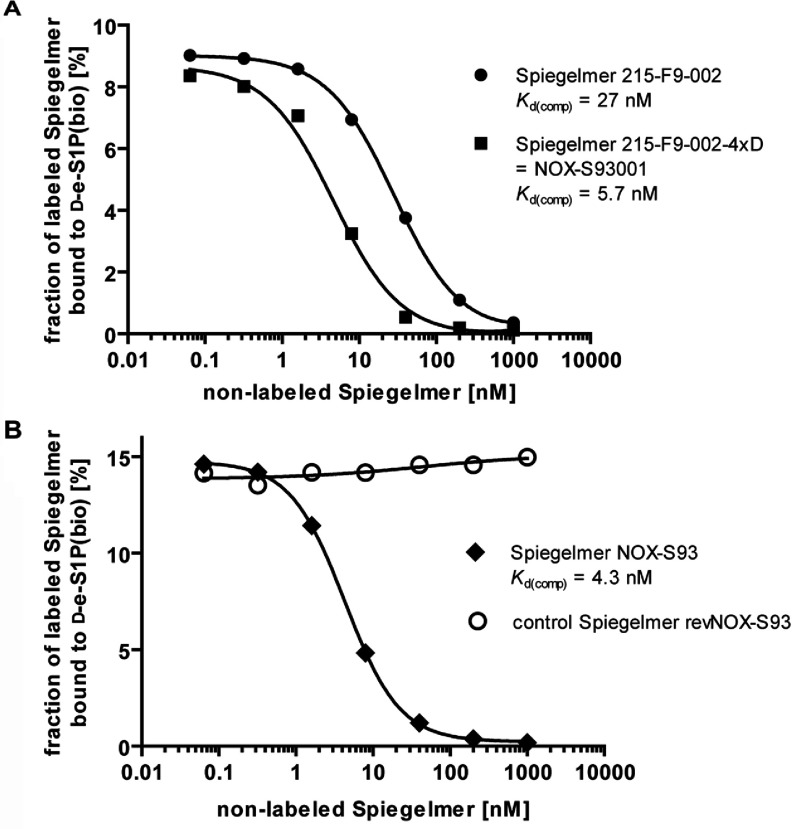
Comparison of equilibrium dissociation constants to S1P of the non-PEGylated Spiegelmers 215-F9–002 compared with 215-F9-002-4xD (NOX-S93001) and the 5′-PEGylated Spiegelmers NOX-S93 compared with revNOX-S93 The affinities were determined in a competitive pull-down assay using labelled 215-F9–002 as a reference. (**A**) 215-F9–002 without modification (●) and with deoxyribonucleotide modifications (■) (215-F9-002-4xD=NOX-S93001). (**B**) NOX-S93 (◆) and the reverse sequence revNOX-S93 (○). The absence of any binding of revNOX-S93 confirmed the specificity of NOX-S93.

An *in vitro* selection process was started with a theoretical library size of more than 3×10^15^ different RNA sequences and concentrations of 10 μM of both library and target S1P [L-e-S1P(bio)]. A relatively high concentration of 4 mg/ml serum albumin was added to the selection buffer to ensure the solubility of S1P. During the first six rounds, no noticeable affinity of the library to the target over background was detectable. Only starting with round seven the ratio of bound RNA compared with the applied concentration of L-e-S1P(bio) slowly increased, indicating emerging affinity in the library (Supplementary Figure S1A at http://www.biochemj.org/bj/462/bj4620153add.htm). In order to select for the best binding aptamers, the stringency of the selection process was gradually increased by decreasing the concentration of both target and library to a final concentration of 40 pM and 2.5 nM respectively, in round 16. The affinity of the enriched library was determined by a competitive pull-down assay [*K*_d(comp)_=88 nM; Supplementary Figure S1B). The bound RNA of round 16 was reverse transcribed, amplified by PCR, cloned and 47 clones were sequenced. The alignment of the clones revealed essentially one sequence with few point mutations (Supplementary Figure S1C). Truncation by omitting the primer binding sites and ranking resulted in the 46mer 215-F9-001. By deleting the terminal A/U base pair of 215-F9-001, a length of 44 nucleotides was defined as the minimal binder (215-F9-002; Supplementary Figure S1C) that displayed a dissociation constant of 53 nM (Supplementary Figure S1D). Further truncations were not possible without loss of affinity. The sequence 215-F9-002 was then synthesized in its L-configuration as a Spiegelmer®. In a competitive pull-down assay the dissociation constant [*K*_d(comp)_] of Spiegelmer® 215-F9-002 to naturally occurring D-e-S1P was determined to be 27 nM (Figure 2A). Variants of 215-F9-002 in which the individual ribonucleotides at positions 1, 11, 19, 21 or 32 are exchanged for the corresponding deoxyribonucleotide revealed improved binding affinity (Figure 1A). The highest affinity increase resulted in a change of positions 21 [*K*_d(comp)_=11 nM] or 19 [*K*_d(comp)_=16 nM]. Combinations of several substitutions were additive and led to 215-F9-002-4xD with four exchanged ribonucleotides and a dissociation constant *K*_d(comp)_ of 5.7 nM (Figure 2A). This 44mer variant constitutes the final candidate sequence (Figure 1A; deoxyribonucleotide positions are shown shaded in grey) named NOX-S93001. A secondary structure prediction using the software *mfold* is depicted in Figure 1(B) [25]. According to this software the whole molecule seems to form a typical hairpin structure with several bulge regions. The respective five terminal nucleotides at each end are self-complementary and thus presumably form a helix.

A test for the stereospecifity of the Spiegelmer® NOX-S93001 and the corresponding aptamer D-NOX-S93001 confirmed the enantioselectivity of this class of molecules. The non-natural enantiomeric L-e-S1P that was used for *in vitro* selection was not bound by the Spiegelmer®, but by the aptamer (Supplementary Figure S2A at http://www.biochemj.org/bj/462/bj4620153add.htm). Conversely, the natural enantiomeric D-e-S1P was bound by the Spiegelmer®, but not by the aptamer (Supplementary Figure S2B). Binding of the Spiegelmer® to D-e-S1P was not dependent on the presence of BSA, which had been used at 4 mg/ml throughout the binding reactions during *in vitro* selection. The Spiegelmer® bound equally well to D-e-S1P irrespective of the presence of 4 mg/ml or of only 0.00012 mg/ml BSA (Supplementary Figure S2C).

Since a PEG-modification that increases the drug's plasma half-life by decreasing renal clearance [26] was deemed to be mandatory for later studies *in vivo*, the Spiegelmer® NOX-S93001 was conjugated to a 40 kDa PEG moiety at its 5′-end and named NOX-S93. The dissociation constant for NOX-S93 [*K*_d(comp)_=4.3 nM] (Figure 2B) was found to be very similar to that of NOX-S93001, demonstrating that PEGylation did not compromise binding affinity. The subsequent *in vitro* studies in cell-based systems were all done with the PEGylated candidate NOX-S93.

### Inhibition of S1P signalling in cell-based assays by NOX-S93

**Figure 3:**
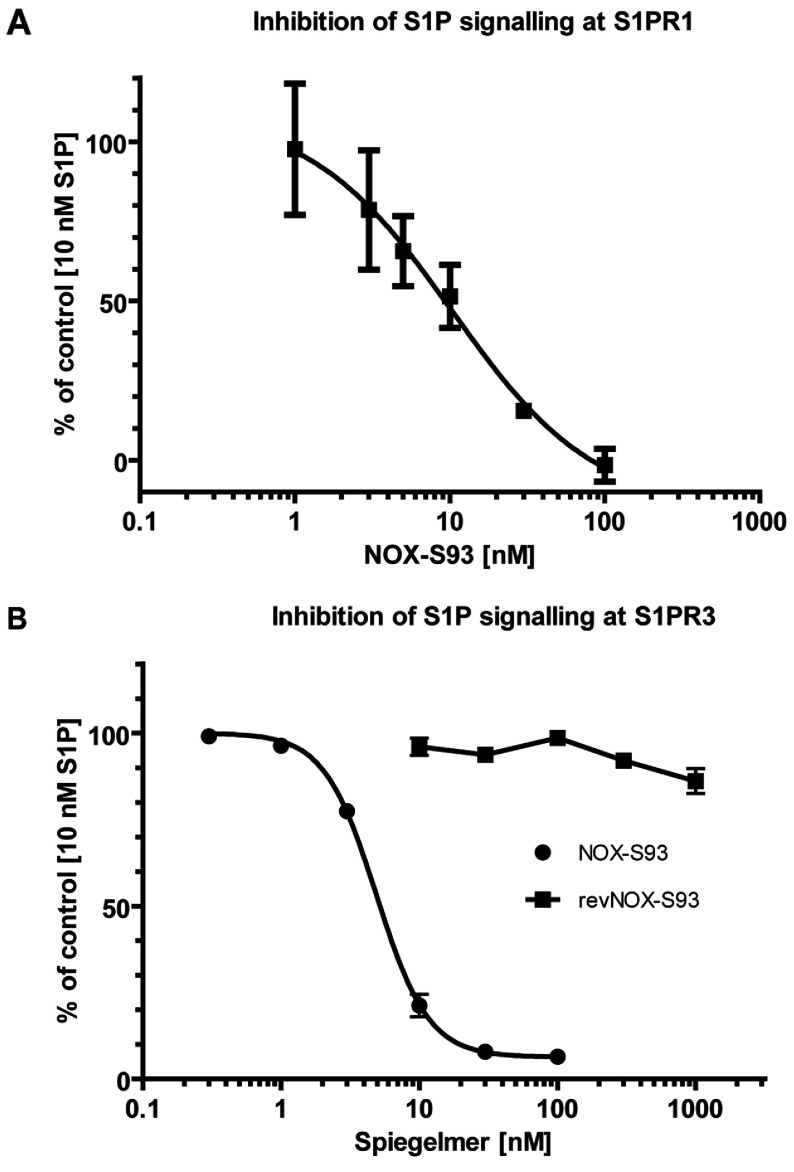
Inhibition of S1P signalling by NOX-S93 in cell-based assays (**A**) PathHunter™ eXpress growth-arrested EDG-1 CHO-K1 β-arrestin cells were stimulated with 10 nM S1P in the presence of the indicated concentrations of NOX-S93. The activity of the reporter enzyme, β-galactosidase, was measured and plotted against the Spiegelmer® concentration as a percentage activity with the largest value set to 100%. In this representative experiment NOX-S93 inhibited the S1PR1 mediated effects of S1P with an IC_50_ of 10.3 nM (means±S.D. of triplicate assays). (**B**) Intracellular calcium signalling in stably transfected CHO cells expressing S1PR3 and G_α15_ was stimulated with 10 nM S1P in the presence of the indicated concentrations of NOX-S93. Increase in intracellular calcium was measured and plotted against the Spiegelmer® concentration as a percentage with the largest value set to 100%. NOX-S93 (●) inhibited the S1P-induced calcium increase with an IC_50_ of 5.45±0.89 nM (*n*=4). The graph is representative of the result of one experiment (means±S.D. of triplicate assays). The control Spiegelmer® revNOX-S93 (■) showed no inhibition (*n*=3).

To confirm that high-affinity binding to S1P also translates into inhibition, the activity of Spiegelmer® NOX-S93 was tested in two cell-based assays employing two different cell lines expressing the human receptors S1PR1 and S1PR3. Signalling in both cell lines was induced by S1P in the same concentration range with EC_50_ values at 10 nM. As shown in Figure 3(A), NOX-S93 inhibited activation of S1PR1 with a low nanomolar IC_50_ (*n*=2). Calcium signalling in the S1PR3-expressing cell line was inhibited by NOX-S93 with an IC_50_ of 5.5 nM (*n*=4) (Figure 3B). The non-functional Spiegelmer® revNOX-S93 with the reverse sequence, tested in the S1PR3-based cell assay, showed no inhibitory effect and confirmed the specificity of the NOX-S93 effect (Figure 3B).

### Cross-reactivity of NOX-S93 to S1P-related lipids

The selectivity of NOX-S93 to bind to S1P was investigated in cell-based assays using structurally related lipids. For this purpose the S1PR3-expressing CHO cell line was used in two different modes. The cross-reactivity to dihydro-, C17- and C20-S1P and lysophosphatidic acid was tested in the direct mode, as these four lipids were able to stimulate intracellular calcium mobilization. All of other tested lipids did not induce S1PR3 signalling. Therefore these other lipids were tested in a different assay format to compete with the inhibitory effect of NOX-S93 on S1P-mediated calcium release. As shown in Table 1, NOX-S93 was only cross-reactive with the most closely related lipids to S1P, namely dihydro-, C17- and C20-S1P, which are inhibited with an IC_50_ of 18, 7.6 and 37 nM respectively. All of other tested lipids were not bound by NOX-S93.

**Table 1:** Selectivity analysis of NOX-S93 with S1P-related lipids The lipids were tested for binding to NOX-S93 in the S1PR3 calcium release assay either by direct inhibition of the S1P-related lipid signalling or by competition of the inhibitory action of 100 nM NOX-S93 on signalling of 10 nM S1P.

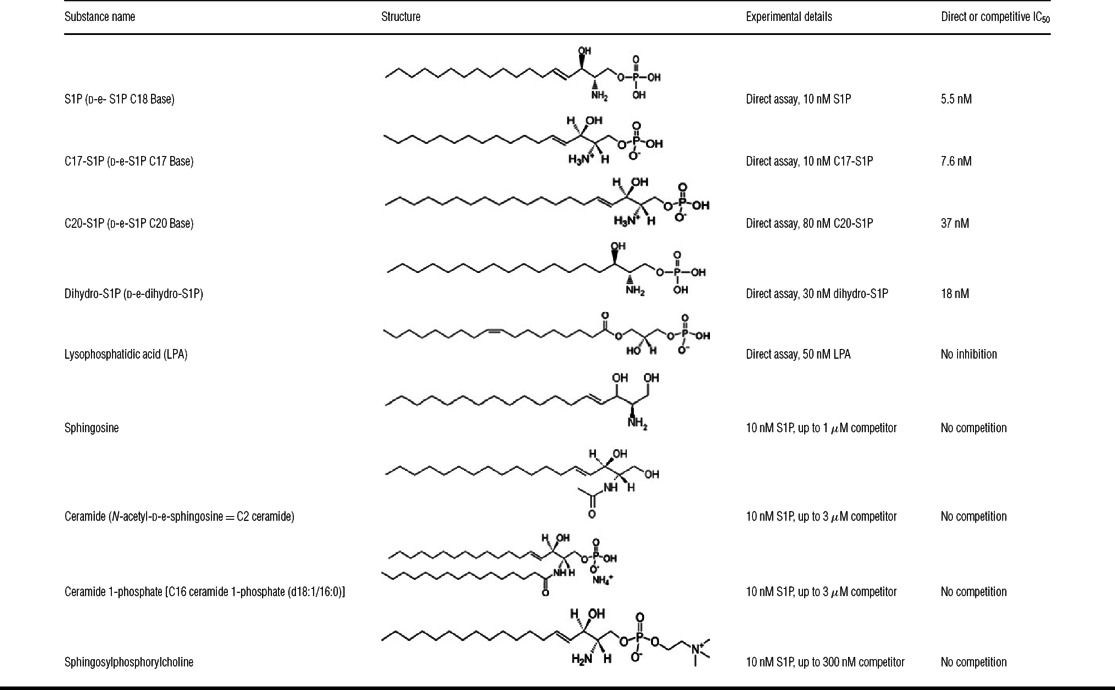

### Efficacy of NOX-S93 in the cellular angiogenesis assay

**Figure 4:**
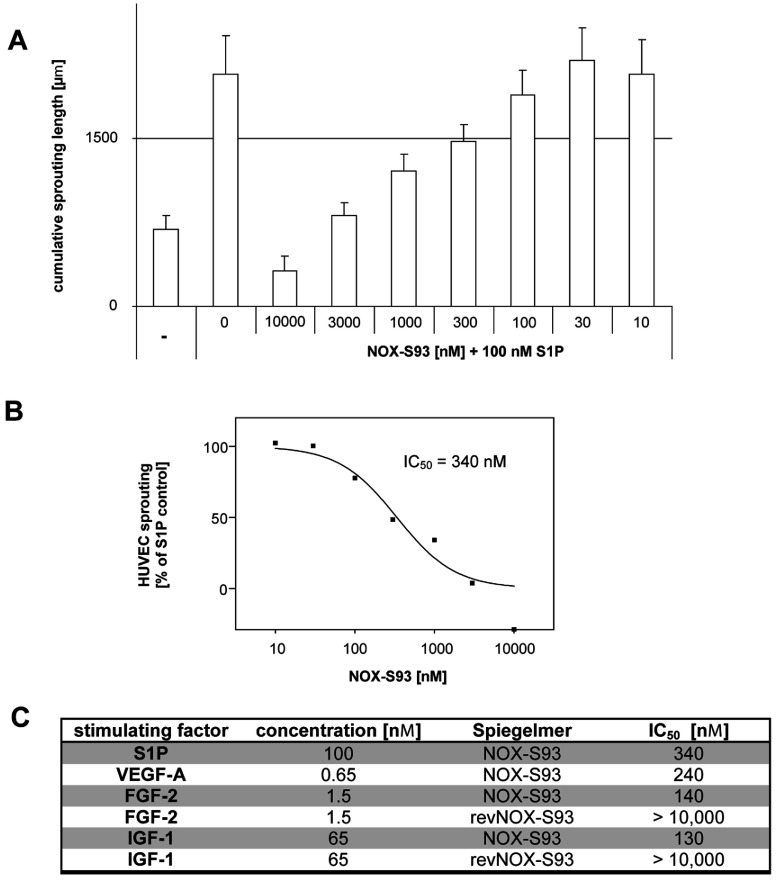
Inhibition of HUVEC spheroid sprouting by NOX-S93 in the cellular angiogenesis assay (**A**) HUVEC spheroids were embedded in a 3D collagen gel, stimulated with 100 nM S1P and incubated for 24 h with different concentrations of NOX-S93. The mean±S.D. of the cumulative sprout length of ten randomly selected spheroids per data point is depicted. −, basal sprouting without S1P stimulation and NOX-S93 treatment as a negative control. (**B**) IC_50_ of NOX-S93 for the inhibition of HUVEC spheroid sprouting after stimulation with 100 nM S1P. (**C**) NOX-S93 IC_50_ values for the inhibition of HUVEC spheroid sprouting after stimulation by different growth factors. The control Spiegelmer® revNOX-S93 showed no inhibition.

The ability of NOX-S93 to inhibit angiogenesis of human endothelial cells was demonstrated in a cellular angiogenesis assay *in vitro*. First, the sprouting of HUVEC spheroids was induced with 100 nM S1P in the presence of different concentrations of NOX-S93. As shown in Figure 4(A), NOX-S93 dose-dependently inhibited the cumulative sprouting length of HUVEC spheroids. The calculated IC_50_ of NOX-S93 for inhibition of S1P-induced sprouting is approximately 340 nM (Figure 4B). At 10000 nM NOX-S93 spheroid sprouting was reduced below negative control levels possibly due to neutralization of basal concentrations of S1P effective in the angiogenesis assay.

Subsequently, it was examined if NOX-S93 could also inhibit angiogenesis that was induced by different human growth factors. For this purpose the sprouting of HUVEC spheroids was induced with VEGF-A, FGF-2 or IGF-1. The sensitivity of the HUVEC spheroids for the applied growth factors revealed to be inhomogeneous with VEGF-A and FGF-2 being approximately 100-fold more potent than IGF-1 or S1P in inducing sprouting. Contrary to this NOX-S93 inhibited each of the four tested pro-angiogenic factors with IC_50_ values in the same range between 130 nM and 340 nM (Figure 4C). The non-functional control Spiegelmer® revNOX-S93 showed no inhibitory effect on HUVEC spheroid sprouting (Figure 4C).

In order to prove the specificity of NOX-S93 regarding the observed inhibition of growth factor induced HUVEC sprouting, we performed competition assays. As shown in Supplementary Figure S3 (http://www.biochemj.org/bj/462/bj4620153add.htm) the growth factors VEGF-A, FGF-2 and IGF-1 did not interfere with NOX-S93-dependent inhibition of S1P in the calcium release assay at concentrations up to 50 nM. These results confirm that the effect of NOX-S93 on growth factor induced HUVEC sprouting shown in Figure 4(C) is not caused by interaction of NOX-S93 with the growth factors, but by inhibition of S1P, indicating that growth factor induced HUVEC sprouting is dependent on S1P signalling.

**Figure 5:**
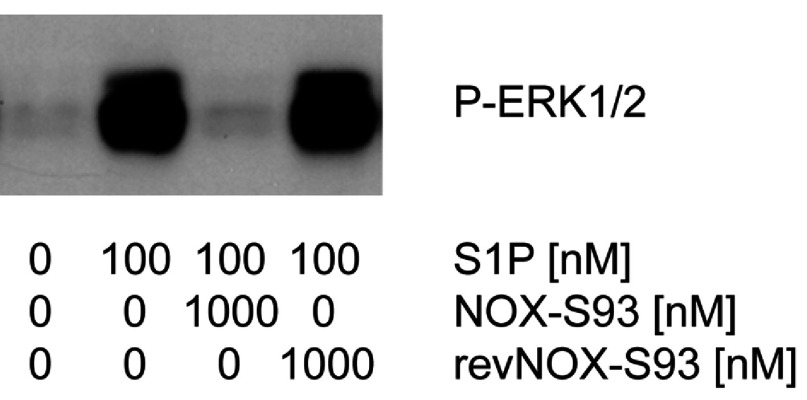
Inhibition of S1P induced ERK1/2 phosphorylation in HUVECs by NOX-S93 HUVECs were cultured in 2D, serum-starved and stimulated with 100 nM S1P in the presence or absence of NOX-S93 or the non-functional control Spiegelmer® revNOX-S93 for 5 min. Activation of ERK1/2 was studied by Western blotting using a phosphospecific antibody. Whereas a 10-fold excess of NOX-S93 completely inhibited S1P-induced ERK1/2 phosphorylation, the control Spiegelmer® revNOX-S93 was inactive.

To show that S1P can exert direct effects on HUVECs which can be inhibited by NOX-S93 we performed Western blot assays and found a strong S1P-induced activation of the ERK1/2 signalling pathway. After 5 min, 100 nM S1P stimulated ERK1/2 phosphorylation in 2D-grown HUVECs. This S1P-induced phosphorylation was completely inhibited by a 10-fold excess of NOX-S93, whereas the non-functional Spiegelmer® revNOX-S93 was inactive (Figure 5).

## DISCUSSION

We describe the identification of a structured mirror-image oligonucleotide, a so-called Spiegelmer®, that binds with high affinity and selectivity to the bioactive sphingolipid S1P thereby inhibiting its action. The target S1P is not only a small molecule (molecular mass of 379 Da) with only limited epitopes for an interaction with a large(r) molecule, but it also carries like other lipids the difficulty of poor solubility in aqueous solutions. This may be the reason why oligonucleotide aptamers capable of binding to lipids are not regularly communicated in the literature. The few examples are bacterial lipopolysaccharide-binding aptamers [27–29], RNA aptamers for the detection of sphingosylphosphorylcholine [30] and aptamers that recognize the lipid moiety of the antibiotic moenomycin A [31].

Before the *in vitro* selection we decided to modify the S1P with a biotin moiety to permit an efficient partitioning of potential target binding from non-binding oligonucleotides. The polar head of S1P containing phosphate, amino and hydroxy groups was expected to be the most likely epitope for an interaction with an oligonucleotide. Therefore the biotin modification was conjugated to the distal end of the selection target to avoid potential sterical hindrance. In order not to compromise the flexibility of the target, however, the biotin was attached via a long linker (AEEAc–AEEAc) to the terminal C18 of the hydrocarbon chain of L-e-S1P.

Neither the starting library nor the pools of the first six selection rounds showed any noticeable affinity to S1P. Not until the seventh round did the RNA pool begin to enrich in S1P-binding aptamers, and after nine more selection rounds the enriched pool was sequenced. The sequencing revealed only one master sequence with a few point mutations (Supplementary Figure S1C). This master sequence could be readily truncated to a minimal binding sequence of 44 nucleotides, named 215-F9-002. This 44mer was then synthesized using the corresponding L-nucleotides to give the Spiegelmer® that was shown to bind to the naturally occurring D-e-S1P (Figure 2A).

After the successful proof that the Spiegelmer® was able to bind to natural S1P, several positions in the RNA sequence were identified that contributed to a higher affinity of the Spiegelmer® following an L-ribonucleotide to L-deoxyribo-nucleotide exchange. Whereas the pure RNA Spiegelmer® binds to S1P with a *K*_d(comp)_ of 27 nM (Figure 2A), at least five single positions were able to increase the affinity up to approximately 2-fold. Finally, a combination of four modifications yielded the candidate 215-F9-002-4xD (NOX-S93001) with an approximately 5-fold-improved dissociation constant. This affinity to S1P is considered potent enough to compete with S1P's reported receptor *K*_d_ values that are in the range of 8 to 50 nM [32].

The 5′-PEGylated Spiegelmer® NOX-S93 was tested for its ability to inhibit S1P in cell-based assays. In the present study we used assay systems that involve the human receptors S1PR1 and S1PR3. We were able to demonstrate that NOX-S93 effectively inhibited S1P signalling at both of the tested S1P receptors in the low nanomolar range, whereas a non-functional control based on the reversed sequence of NOX-S93 did not show any inhibition. We deduce from these findings that NOX-S93 most likely will also inhibit S1P signalling at the remaining three S1P receptors.

In an additional experimental setting we investigated the selectivity of NOX-S93 with a panel of related lipids (Table 1). As already expected, the polar head of S1P was found to be essential for binding since none of the tested S1P-related lipids with slightly different polar head groups was recognized by the Spiegelmer®. Only dihydro-S1P, C17-S1P and C20-S1P, the most closely related lipids to S1P with exactly the same polar head, were well bound displaying IC_50_ values of 18, 7.6 and 37 nM respectively. However the cells had to be stimulated with 30 nM dihydro-S1P, 10 nM C17-S1P and 80 nM C20-S1P to achieve a cellular signal comparable with 10 nM S1P. Thus IC_50_ values partly appeared a bit weaker presumably due to stoichiometric reasons and the Spiegelmer® affinity to these three lipids is considered to be very similar to S1P. Since shortening (C17-S1P) or elongating (C20-S1P) the hydrocarbon chain do not significantly influence the binding of NOX-S93, the hydrocarbon chain apparently is not as important for binding as the polar head group. Nevertheless it cannot be excluded that in addition to the polar head group the hydrocarbon chain or a part of it contributes to the binding region.

As demonstrated, we found that NOX-S93 does not bind to the S1P-related sphingolipids ceramide and sphingosine. This may be of particular importance for the therapeutic intervention in cancer indications where the balance between S1P as a mediator of cell proliferation and its precursors, ceramide and sphingosine as growth arrest and apoptosis-promoting sphingolipids is disturbed by increased S1P or decreased sphingosine and/or ceramide levels [3]. In a therapeutic intervention, NOX-S93, specifically binding and neutralizing S1P without affecting ceramide and sphingosine, could possibly switch the balance from proliferation to apoptosis of tumour cells. An S1P-neutralizing monoclonal antibody has already demonstrated its anti-tumorigenic potential in mouse tumour models [6,11].

The capillary tube formation of endothelial cells as an early process of blood vessel formation is induced by S1P in a comparable manner with other pro-angiogenic growth factors such as VEGF-A, FGF-2 or IGF-1 [33–35]. *In vitro* studies have shown that growth factor signalling leads to increased S1P release by the phosphorylation and subsequent translocation of SK1 to the inner side of the cell membrane where its substrate, sphingosine, is located [36,37]. *In vivo*, the central role of S1P in angiogenesis became clear when it was shown that SK1 and SK2-double-knockout mice died *in utero* due to defects in neuroangiogenesis and angiogenesis [38] as did S1PR-knockout animals owing to severe haemorrhaging and incomplete vasculogenesis [39]. In our *in vitro* assays we were able to demonstrate that NOX-S93 blocks the pro-angiogenic action not only of S1P, but also of the abovementioned growth factors in a cellular 3D angiogenesis assay with HUVECs. The unexpectedly high IC_50_ value of NOX-S93 in our experimental setting may be explained with the relatively high S1P concentration (100 nM) needed to elicit the angiogenic effect. We suppose that similar S1P concentrations are locally effective in the growth factor-stimulated HUVEC sprouting assays. NOX-S93, being a ligand-neutralizing agent and not a receptor blocker, needs to be present in excess over S1P itself and therefore needs to be present in high concentrations allowing for free NOX-S93 to be present during the whole assay time.

A bidirectional cross-talk between VEGF and S1P/S1PR1 is well known. S1P increases the expression of *VEGF* mRNA in HUVECs, presumably by enhancing the *VEGF* promoter activity [40] and, vice versa, VEGF up-regulates S1PR1 expression and enhances S1P-mediated signalling [41]. This provides a mechanistic explanation for the inhibition of VEGF-induced sprouting of HUVEC spheroids by NOX-S93. The VEGF-induced enhancement of S1PR1 signalling by S1PR1 up-regulation could not come into effect, whereas the ligand for S1PR1 was neutralized by NOX-S93. Our observation is in agreement with the action of an S1P-binding monoclonal antibody that also inhibits the action of VEGF-A and FGF-2 on vessel formation by endothelial cells [11].

In HUVECs we demonstrated ERK1/2 phosphorylation after stimulation by S1P. It has been long known that the ERK signalling pathway plays an important role in endothelial cells during angiogenesis [42–44]. After we could show that S1P driven *in vitro* angiogenesis was inhibited by NOX-S93 it was evident that NOX-S93 was able to inhibit S1P induced ERK phosphorylation in HUVECs.

S1P possesses chemotactic potential [34]. As the pro-angiogenic effect of IGF-1 has been demonstrated to be associated with the induction of endothelial cell migration [45], we hypothesized that this effect could be mediated by S1P. In our experimental setting we demonstrate for the first time that IGF-1-induced angiogenesis can indeed be inhibited by neutralization of S1P. Thus extracellular S1P signalling may be a general prerequisite for cell motility, which is central for the formation of ordered structures such as blood vessels. In agreement with this concept, a recent study indicated that NOX-S93 inhibits the motility of human rhabdomyosarcoma cells *in vitro* and prevents radio/chemotherapy-induced metastasis *in vivo* [46]. Our results together with these positive *in vivo* data encourage further studies to test the capability of NOX-S93 as a novel therapeutic in disease patterns related to angiogenesis and S1P.

## Online data

Supplementary data

## References

[1] Spiegel S., Milstien S. (2003). Sphingosine-1-phosphate: an enigmatic signalling lipid. Nat. Rev. Mol. Cell Biol..

[2] Furuya H., Shimizu Y., Kawamori T. (2011). Sphingolipids in cancer. Cancer Metastasis Rev..

[3] Pyne N. J., Pyne S. (2010). Sphingosine 1-phosphate and cancer. Nat. Rev. Cancer..

[4] Spiegel S., Milstien S. (2000). Functions of a new family of sphingosine-1-phosphate receptors. Biochim. Biophys. Acta.

[5] Sabbadini R. A. (2011). Sphingosine-1-phosphate antibodies as potential agents in the treatment of cancer and age-related macular degeneration. Br. J. Pharmacol..

[6] Ponnusamy S., Selvam S. P., Mehrotra S., Kawamori T., Snider A. J., Obeid L. M., Shao Y., Sabbadini R., Ogretmen B. (2012). Communication between host organism and cancer cells is transduced by systemic sphingosine kinase 1/sphingosine 1-phosphate signalling to regulate tumour metastasis. EMBO Mol. Med..

[7] Gellings Lowe N., Swaney J. S., Moreno K. M., Sabbadini R. A. (2009). Sphingosine-1-phosphate and sphingosine kinase are critical for transforming growth factor-β-stimulated collagen production by cardiac fibroblasts. Cardiovasc. Res..

[8] Cyster J. G., Schwab S. R. (2012). Sphingosine-1-phosphate and lymphocyte egress from lymphoid organs. Annu. Rev. Immunol..

[9] Brinkmann V., Billich A., Baumruker T., Heining P., Schmouder R., Francis G., Aradhye S., Burtin P. (2010). Fingolimod (FTY720): discovery and development of an oral drug to treat multiple sclerosis. Nat. Rev. Drug Discov..

[10] Dickson M. A., Carvajal R. D., Merrill A. H., Gonen M., Cane L. M., Schwartz G. K. (2011). A phase I clinical trial of safingol in combination with cisplatin in advanced solid tumors. Clin. Cancer Res..

[11] Visentin B., Vekich J. A., Sibbald B. J., Cavalli A. L., Moreno K. M., Matteo R. G., Garland W. A., Lu Y., Yu S., Hall H. S. (2006). Validation of an anti-sphingosine-1-phosphate antibody as a potential therapeutic in reducing growth, invasion, and angiogenesis in multiple tumor lineages. Cancer Cell.

[12] O’Brien N., Jones S. T., Williams D. G., Cunningham H. B., Moreno K., Visentin B., Gentile A., Vekich J., Shestowsky W., Hiraiwa M. (2009). Production and characterization of monoclonal anti-sphingosine-1-phosphate antibodies. J. Lipid Res..

[13] Tuerk C., Gold L. (1990). Systematic evolution of ligands by exponential enrichment: RNA ligands to bacteriophage T4 DNA polymerase. Science.

[14] Ellington A. D., Szostak J. W. (1990). *In vitro* selection of RNA molecules that bind specific ligands. Nature.

[15] Klussmann S., Nolte A., Bald R., Erdmann V. A., Furste J. P. (1996). Mirror-image RNA that binds D-adenosine. Nat. Biotechnol..

[16] Vater A., Sell S., Kaczmarek P., Maasch C., Buchner K., Pruszynska-Oszmalek E., Kolodziejski P., Purschke W. G., Nowak K. W., Strowski M. Z., Klussmann S. (2013). A mixed mirror-image DNA/RNA aptamer inhibits glucagon and acutely improves glucose tolerance in models of type 1 and type 2 diabetes. J. Biol. Chem..

[17] Denekas T., Troltzsch M., Vater A., Klussmann S., Messlinger K. (2006). Inhibition of stimulated meningeal blood flow by a calcitonin gene-related peptide binding mirror-image RNA oligonucleotide. Br. J. Pharmacol..

[18] Purschke W. G., Eulberg D., Buchner K., Vonhoff S., Klussmann S. (2006). An L-RNA-based aquaretic agent that inhibits vasopressin *in vivo*. Proc. Natl. Acad. Sci. U.S.A..

[19] Hoehlig K., Maasch C., Shushakova N., Buchner K., Huber-Lang M., Purschke W. G., Vater A., Klussmann S. (2013). A Novel C5a-neutralizing mirror-image L-aptamer prevents organ failure and improves survival in experimental sepsis. Mol. Ther..

[20] Darisipudi M. N., Kulkarni O. P., Sayyed S. G., Ryu M., Migliorini A., Sagrinati C., Parente E., Vater A., Eulberg D., Klussmann S. (2011). Dual blockade of the homeostatic chemokine CXCL12 and the proinflammatory chemokine CCL2 has additive protective effects on diabetic kidney disease. Am. J. Pathol..

[21] Eulberg D., Purschke W., Anders H. J., Selve N., Klussmann S., Kurreck J. (2008). Spiegelmer NOX-E36 for renal diseases. Therapeutic Oligonucleotides.

[22] Schwoebel F., van Eijk L. T., Zboralski D., Sell S., Buchner K., Maasch C., Purschke W. G., Humphrey M., Zollner S., Eulberg D. (2013). The effects of the anti-hepcidin Spiegelmer NOX-H94 on inflammation-induced anemia in cynomolgus monkeys. Blood.

[23] Hoffmann S., Hoos J., Klussmann S., Vonhoff S. (2011). RNA aptamers and spiegelmers: synthesis, purification, and post-synthetic PEG conjugation. Curr. Protoc. Nucleic Acid Chem..

[24] Korff T., Augustin H. G. (1998). Integration of endothelial cells in multicellular spheroids prevents apoptosis and induces differentiation. J. Cell Biol..

[25] Zuker M. (2003). Mfold web server for nucleic acid folding and hybridization prediction. Nucleic Acids Res..

[26] Keefe A. D., Pai S., Ellington A. (2010). Aptamers as therapeutics. Nat. Rev. Drug Discov..

[27] Ding J. L., Gan S. T., Ho B. (2009). Single-stranded DNA oligoaptamers: molecular recognition and LPS antagonism are length- and secondary structure-dependent. J. Innate Immun..

[28] Lee Y. J., Han S. R., Maeng J. S., Cho Y. J., Lee S. W. (2012). *In vitro* selection of *Escherichia coli* O157:H7-specific RNA aptamer. Biochem. Biophys. Res. Commun..

[29] Wen A. Q., Yang Q. W., Li J. C., Lv F. L., Zhong Q., Chen C. Y. (2009). A novel lipopolysaccharide-antagonizing aptamer protects mice against endotoxemia. Biochem. Biophys. Res. Commun..

[30] Horii K., Omi K., Yoshida Y., Imai Y., Sakai N., Oka A., Masuda H., Furuichi M., Tanimoto T., Waga I. (2010). Development of a sphingosylphosphorylcholine detection system using RNA aptamers. Molecules.

[31] Betat H., Vogel S., Struhalla M., Forster H. H., Famulok M., Welzel P., Hahn U. (2003). Aptamers that recognize the lipid moiety of the antibiotic moenomycin A. Biol. Chem..

[32] Chun J., Rosen H. (2006). Lysophospholipid receptors as potential drug targets in tissue transplantation and autoimmune diseases. Curr. Pharm. Des..

[33] Ferrara N. (2004). Vascular endothelial growth factor: basic science and clinical progress. Endocr. Rev..

[34] Lee O. H., Kim Y. M., Lee Y. M., Moon E. J., Lee D. J., Kim J. H., Kim K. W., Kwon Y. G. (1999). Sphingosine 1-phosphate induces angiogenesis: its angiogenic action and signaling mechanism in human umbilical vein endothelial cells. Biochem. Biophys. Res. Commun..

[35] Dunn S. E. (2000). Insulin-like growth factor I stimulates angiogenesis and the production of vascular endothelial growth factor. Growth Horm. IGF Res..

[36] Stahelin R. V., Hwang J. H., Kim J. H., Park Z. Y., Johnson K. R., Obeid L. M., Cho W. (2005). The mechanism of membrane targeting of human sphingosine kinase 1. J. Biol. Chem..

[37] Takabe K., Paugh S. W., Milstien S., Spiegel S. (2008). “Inside-out” signaling of sphingosine-1-phosphate: therapeutic targets. Pharmacol. Rev..

[38] Mizugishi K., Yamashita T., Olivera A., Miller G. F., Spiegel S., Proia R. L. (2005). Essential role for sphingosine kinases in neural and vascular development. Mol. Cell Biol..

[39] Liu Y., Wada R., Yamashita T., Mi Y., Deng C. X., Hobson J. P., Rosenfeldt H. M., Nava V. E., Chae S. S., Lee M. J., Liu C. H. (2000). Edg-1, the G protein-coupled receptor for sphingosine-1-phosphate, is essential for vascular maturation. J. Clin. Invest..

[40] Heo K., Park K. A., Kim Y. H., Kim S. H., Oh Y. S., Kim I. H., Ryu S. H., Suh P. G. (2009). Sphingosine 1-phosphate induces vascular endothelial growth factor expression in endothelial cells. BMB Rep..

[41] Igarashi J., Erwin P. A., Dantas A. P., Chen H., Michel T. (2003). VEGF induces S1P1 receptors in endothelial cells: Implications for cross-talk between sphingolipid and growth factor receptors. Proc. Natl. Acad. Sci. U.S.A..

[42] Eliceiri B. P., Klemke R., Stromblad S., Cheresh D. A. (1998). Integrin αvβ3 requirement for sustained mitogen-activated protein kinase activity during angiogenesis. J. Cell Biol..

[43] Giroux S., Tremblay M., Bernard D., Cardin-Girard J. F., Aubry S., Larouche L., Rousseau S., Huot J., Landry J., Jeannotte L., Charron J. (1999). Embryonic death of Mek1-deficient mice reveals a role for this kinase in angiogenesis in the labyrinthine region of the placenta. Curr. Biol..

[44] Meadows K. N., Bryant P., Pumiglia K. (2001). Vascular endothelial growth factor induction of the angiogenic phenotype requires Ras activation. J. Biol. Chem..

[45] Su E. J., Cioffi C. L., Stefansson S., Mittereder N., Garay M., Hreniuk D., Liau G. (2003). Gene therapy vector-mediated expression of insulin-like growth factors protects cardiomyocytes from apoptosis and enhances neovascularization. Am. J. Physiol. Heart Circ. Physiol..

[46] Schneider G., Bryndza E., Abdel-Latif A., Ratajczak J., Maj M., Tarnowski M., Klyachkin Y. M., Houghton P., Morris A. J., Vater A. (2013). Bioactive lipids S1P and C1P are prometastatic factors in human rhabdomyosarcoma, and their tissue levels increase in response to radio/chemotherapy. Mol. Cancer Res..

