# Supplementary material for: Identification and characterization of a mirror-image oligonucleotide that binds and neutralizes sphingosine 1-phosphate, a central mediator of angiogenesis
Source: Biochem J. 2014 Jul 24;462(Pt 1):153–62. doi: 10.1042/BJ20131422 (PMC4109837; doi:10.1042/BJ20131422)
Supplement: Supplementary data [file bj4620153add.pdf]

Biochem. J. (2014) **462**, 153–162 (Printed in Great Britain) doi:10.1042/BJ20131422

SUPPLEMENTARY ONLINE DATA

Identification and characterization of a mirror-image oligonucleotide that binds and neutralizes sphingosine 1-phosphate, a central mediator of angiogenesis

Werner G. PURSCHKE*[1,2], Kai HOEHLIG*[2], Klaus BUCHNER*[2], Dirk ZBORALSKI*[2], Frank SCHWOEBEL*[2], Axel VATER*[2] and Sven KLUSSMANN*[2]

*NOXXON Pharma AG, Berlin, Germany

Supplementary Figures S1–S3 can be found on the following pages.

[1] To whom correspondence should be addressed (email wpurschke@noxxon.com).
[2] All of the authors are employees of NOXXON Pharma AG, which has filed patents on the S1P-neutralizing Spiegelmer®.

© 2014 The Author(s)
This is an Open Access article distributed under the terms of the Creative Commons Attribution Licence (CC-BY) (http://creativecommons.org/licenses/by/3.0/) which permits unrestricted use, distribution and reproduction in any medium, provided the original work is properly cited.

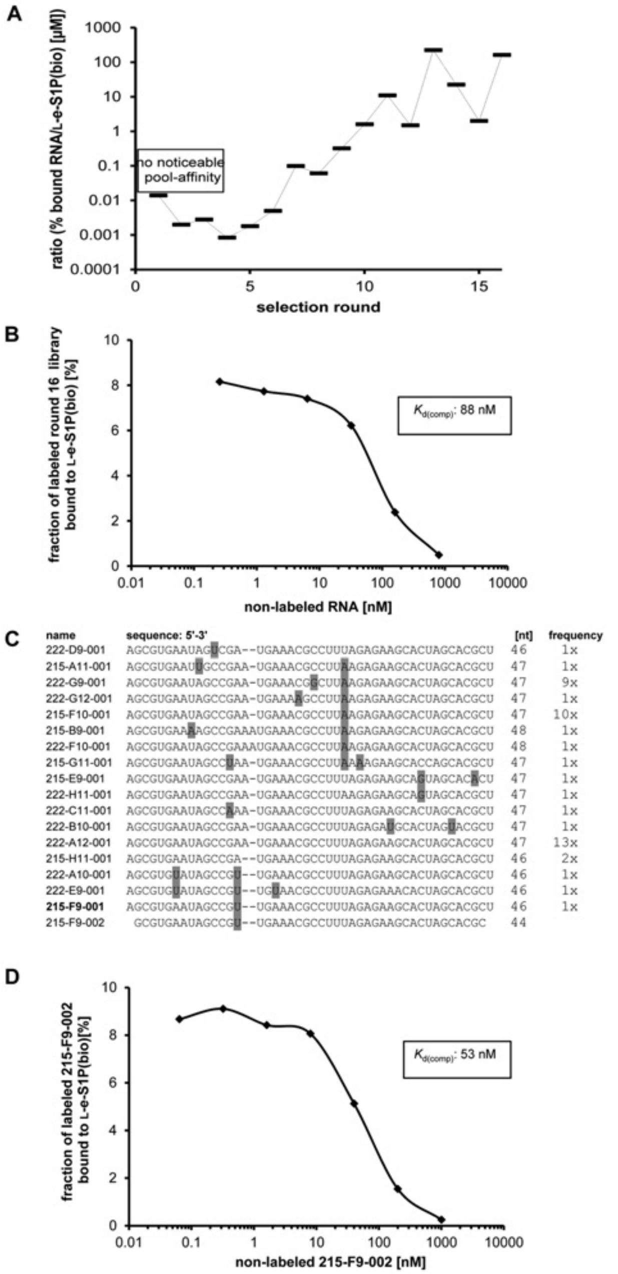

Figure S1 Enrichment and characterization of RNA binders to biotinylated L-e-S1P

(**A**) As an indicator for an increase in library affinity the ratio of the RNA library's fraction bound to the target as a percentage (%) and the concentration of L-e-S1P(bio) (μM) was calculated and plotted against the selection round number. (**B**) $K_{d(comp)}$ value of the library in round 16, determined by a competitive pull-down assay. (**C**) The alignment of 47 sequenced clones essentially revealed one sequence with few point mutations (grey). By omitting the primer binding sites the 46mer 215-F9-001 was defined and further truncated to the 44mer 215-F9-002. (**D**) $K_{d(comp)}$ value of the 44mer 215-F9-002, determined by a competitive pull-down assay.

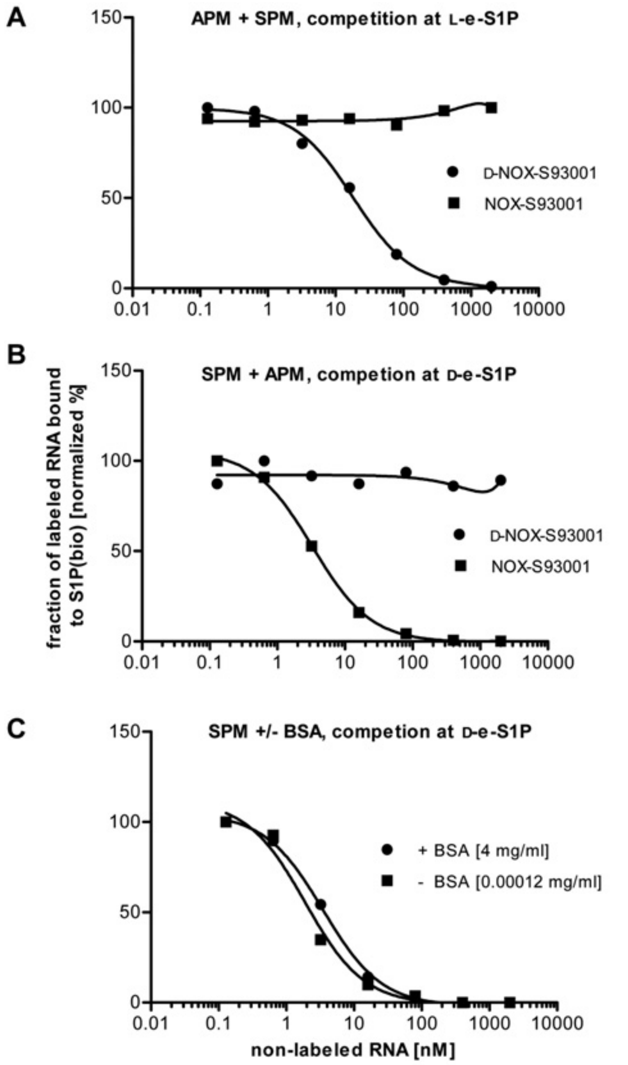

Figure S2 Stereospecificity of the S1P-binding Spiegelmer®/aptamer and influence of BSA on binding

(**A**) Radioactively labelled aptamer D-NOX-S93001 bound to biotinylated L-e-S1P was competed by increasing amounts of non-labelled aptamer, but not by increasing amounts of non-labeled Spiegelmer®. (**B**) Radioactively labelled Spiegelmer® NOX-S93001 bound to biotinylated D-e-S1P was competed by increasing amounts of non-labelled Spiegelmer®, but not by increasing amounts of non-labelled aptamer. (**C**) Radioactively labelled Spiegelmer NOX-S93001, bound to 3 nM biotinylated D-e-S1P, was equally well competed by increasing amounts of non-labelled Spiegelmer® irrespective of the BSA concentration.

© 2014 The Author(s)
This is an Open Access article distributed under the terms of the Creative Commons Attribution Licence (CC-BY) (http://creativecommons.org/licenses/by/3.0/) which permits unrestricted use, distribution and reproduction in any medium, provided the original work is properly cited.

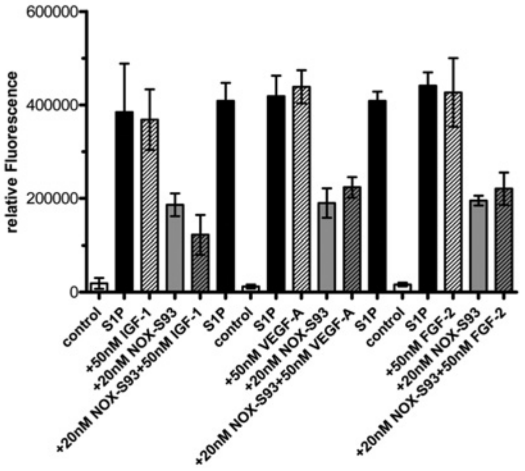

Figure S3 Specificity of NOX-S93 mode of action

Intracellular calcium release in stably transfected CHO cells expressing S1PR3 and $G_{\alpha 15}$ was stimulated with 10 nM S1P in the presence or absence of 20 nM NOX-S93 and 50 nM of the growth factors used in the HUVEC sprouting assay. The graph shows the result of one out of three experiments with very similar results. NOX-S93-induced inhibition of S1P-mediated calcium release was not influenced by the growth factors VEGF-A, IGF-1 and FGF-2 confirming the specificity of NOX-S93. Results are means$\pm$S.D. of assays performed in triplicate.

Received 28 October 2013/15 April 2014; accepted 16 May 2014
Published as BJ Immediate Publication 16 May 2014, doi:10.1042/BJ20131422

© 2014 The Author(s)
This is an Open Access article distributed under the terms of the Creative Commons Attribution Licence (CC-BY) (http://creativecommons.org/licenses/by/3.0/) which permits unrestricted use, distribution and reproduction in any medium, provided the original work is properly cited.
